# Protein–ligand complex structure from serial femtosecond crystallography using soaked thermolysin microcrystals and comparison with structures from synchrotron radiation

**DOI:** 10.1107/S2059798317008919

**Published:** 2017-07-31

**Authors:** Hisashi Naitow, Yoshinori Matsuura, Kensuke Tono, Yasumasa Joti, Takashi Kameshima, Takaki Hatsui, Makina Yabashi, Rie Tanaka, Tomoyuki Tanaka, Michihiro Sugahara, Jun Kobayashi, Eriko Nango, So Iwata, Naoki Kunishima

**Affiliations:** a Bio-Specimen Platform Group, RIKEN SPring-8 Center, Kouto, Sayo-cho, Sayo-gun, Hyogo 679-5148, Japan; b XFEL Research and Development Division, RIKEN SPring-8 Center, Kouto, Sayo-cho, Sayo-gun, Hyogo 679-5148, Japan; c Japan Synchrotron Radiation Research Institute, Kouto, Sayo-cho, Sayo-gun, Hyogo 679-5148, Japan; d SACLA Science Research Group, RIKEN SPring-8 Center, Kouto, Sayo-cho, Sayo-gun, Hyogo 679-5148, Japan

**Keywords:** X-ray free-electron laser, diffraction before destruction, microcrystal, thermolysin, structure-based drug design, X-ray crystallography

## Abstract

Serial femtosecond crystallography (SFX) with an X-ray free-electron laser is used for the structural determination of proteins from a large number of microcrystals at room temperature. To examine the feasibility of pharmaceutical applications of SFX, a ligand-soaking experiment using thermolysin microcrystals has been performed using SFX. The results were compared with those from a conventional experiment with synchrotron radiation (SR) at 100 K. A protein–ligand complex structure was successfully obtained from an SFX experiment using microcrystals soaked with a small-molecule ligand; both oil-based and water-based crystal carriers gave essentially the same results. In a comparison of the SFX and SR structures, clear differences were observed in the unit-cell parameters, in the alternate conformation of side chains, in the degree of water coordination and in the ligand-binding mode.

## Abstract 2

The applicability of the ligand-soaking method in serial femtosecond crystallography has been examined to examine the feasibility of pharmaceutical applications of X-ray free-electron lasers.

## 1. Introduction   

X-ray free-electron lasers (XFELs) generate very short/intense pulses that enable the collection of diffraction data before the destruction of the specimen (Neutze *et al.*, 2000 ▸). This ‘diffraction-before-destruction’ principle of XFELs has successfully been applied in serial femtosecond crystallo­graphy (SFX), in which hundreds of thousands of single-shot diffraction images from randomly oriented microcrystals at room temperature are merged to determine a crystal structure (Chapman *et al.*, 2011 ▸; Boutet *et al.*, 2012 ▸). To date, a substantial number of SFX structures have been reported, including those of natively inhibited trypanosome protease from *in vivo*-grown microcrystals (Redecke *et al.*, 2013 ▸), of membrane proteins from microcrystals grown in lipidic cubic phase (Zhang *et al.*, 2015 ▸; Kang *et al.*, 2015 ▸) and of the photoactive yellow protein in a time-resolved pump–probe experiment (Pande *et al.*, 2016 ▸). Because SFX provides crystal structures at room temperature without radiation damage, it has the potential to be a useful tool in structural biology, which requires structural information under physiological conditions. For instance, a damage-free structure from SFX could account for the proton-transfer mechanism of nitrite reductase (Fukuda *et al.*, 2016 ▸). From this point of view, structure-based drug design (SBDD) is expected to be a likely application of SFX (Zhang *et al.*, 2015 ▸; Hol, 2015 ▸). In SBDD, a small-molecule ligand is designed so as to improve its affinity for the target protein based on the structure of protein–ligand complex crystals, which are typically prepared by soaking protein crystals into a solution containing the ligand (Hol, 1986 ▸; Klebe, 2000 ▸). However, the applicability of soaked crystals in SFX has not fully been examined to date. Here, we present a ligand-soaking experiment in SFX using microcrystals of thermolysin, which has recently been demonstrated as a model system (Hattne *et al.*, 2014 ▸). From a comparison of the SFX structures with those of a conventional experiment using synchrotron radiation at low temperatures, the applicability of SFX to SBDD will be discussed.

## 2. Materials and methods   

### 2.1. Sample preparation   

Lyophilized thermolysin powder from *Bacillus stearo­thermophilus* (Hampton Research) was solubilized in 50 m*M* NaOH in water. Microcrystals of thermolysin were prepared as reported previously (Hattne *et al.*, 2014 ▸) with slight modifications. Crystallization was performed by a batch method on a 50 µl scale; equal volumes (25 µl each) of the thermolysin solution at a concentration of 42.5 mg ml^−1^ and a reservoir solution comprising 40% PEG 2000 MME, 0.1 *M* MES–NaOH pH 6.5, 5 m*M* CaCl_2_ were mixed and incubated at 277 K for 5 h. Elliptical-shaped microcrystals grew to approximate dimensions of 4 × 4 × 8 µm. After the batch crystallization, the microcrystals were collected by centrifugation at 3000*g*, suspended in 500 µl harvest solution comprising 20% PEG 2000 MME, 0.1 *M* MES–NaOH pH 6.5, 5 m*M* CaCl_2_ and filtered through a mesh with a 50 µm pore size. To remove a copurified ligand (Birrane *et al.*, 2014 ▸), the microcrystal suspension was incubated at room temperature for 24 h (back-soaking). The back-soaked microcrystals were collected by centrifugation and resuspended in the harvest solution for the unliganded oil–SFX form, whereas they were resuspended in a soaking solution comprising 20% PEG 2000 MME, 60 m*M* *N*-carbobenzoxy-l-aspartic acid (ZA), 0.1 *M* MES–NaOH pH 6.5, 5 m*M* CaCl_2_ for the liganded oil/water–SFX forms. The soaking samples were incubated at room temperature for 48 h (soaking) and the microcrystals were collected by centrifugation and resuspended in the soaking solution. After back-soaking or soaking, the suspensions contained about 10^8^ microcrystals per millilitre. Because a 1:9 mixture of the microcrystal suspension and the crystal carrier (oil-based or water-based) was used in the SFX experiment (Sugahara *et al.*, 2015 ▸, 2016 ▸), the final specimen contained about 10^7^ microcrystals per millilitre. For the liganded/unliganded oil–SFX forms and the liganded water–SFX form, the synthetic grease Super Lube (Synco Chemical) and an aqueous solution of 12% hydroxyethyl cellulose (Sigma) containing 20% PEG 2000 MME, 60 m*M* ZA, 50 m*M* MES–NaOH pH 6.5 and 2.5 m*M* CaCl_2_ were used as the crystal carrier, respectively. The details of cellulose as a water-based crystal carrier for SFX will be published elsewhere. Conventional macrocrystals soaked with ligand were prepared as reported by Birrane *et al.* (2014 ▸) with slight modifications. Crystallization was performed by the hanging-drop vapour-diffusion method at 293 K using protein solution at a concentration of 25 mg ml^−1^ and a reservoir solution comprising 10% PEG 2000 MME, 0.1 *M* MES–NaOH pH 6.5, 5 m*M* CaCl_2_. From a 2 µl crystallization drop prepared by mixing equal volumes of protein solution and reservoir solution, hexagonal crystals grew in 5 d to approximate dimensions of 60 × 60 × 150 µm. The crystals were back-soaked for 48 h and then soaked for 24 h at room temperature using the same solutions as used for the microcrystals apart from a reduced ZA concentration of 30 m*M* in the soaking solution. The soaked macrocrystals were flash-cooled in liquid nitrogen: after treatment with a cryoprotectant solution [30%(*v*/*v*) PEG 400, 14% PEG 2000 MME, 30 m*M* ZA, 70 m*M* MES–NaOH pH 6.5, 3.5 m*M* CaCl_2_] for the liganded SR1 form and as is for the liganded SR2 form (20% PEG 2000 MME, 30 m*M* ZA, 0.1 *M* MES–NaOH pH 6.5, 5 m*M* CaCl_2_). Although faint ice rings were observed in the diffraction images of the liganded SR2 form, the data were acceptable for structure determination, as shown later.

### 2.2. X-ray data collection and structure determination   

SFX data at room temperature (about 300 K) were collected using a custom-built multi-port CCD detector (MPCCD; Kameshima *et al.*, 2014 ▸) on beamline BL3 at SACLA, Japan (Ishikawa *et al.*, 2012 ▸; Tono *et al.*, 2013 ▸). The parameters of the XFEL beam used were a wavelength of 1.771 Å with about 0.1% standard deviation, a repetition rate of 30 Hz, a temporal width of about 10 fs (FWHM) and a pulse energy of about 450 µJ at the light source (about 200 µJ at the sample). The XFEL beam was focused to 1.5 × 1.5 µm using Kirkpatrick–Baez mirrors (Yumoto *et al.*, 2013 ▸). An aluminium attenuator with a thickness of 50 µm was used to prevent saturation from strong reflections. The sample-to-detector distance was set to 51.5 mm. The SFX experiment was performed using the DAPHNIS chamber with a humid helium ambience (Tono *et al.*, 2015 ▸). The microcrystals suspended in an oil-based or water-based crystal carrier were loaded to the interaction region with XFEL pulses using the syringe-injector system as described in Sugahara *et al.* (2015 ▸, 2016 ▸); the inner diameter of the needle used and the sample flow rate were 110 µm and 0.48 µl min^−1^, respectively. Image data from SFX were retrieved using the SACLA data-acquisition system (Joti *et al.*, 2015 ▸) with filtering by *Cheetah* (Barty *et al.*, 2014 ▸; Nakane *et al.*, 2016 ▸) to extract images containing Bragg spots. The SFX data were processed and scaled using *CrystFEL* v.0.6.0 (White *et al.*, 2012 ▸) without a σ cutoff. The processed data did not include overloaded reflections. Unit-cell parameters were analyzed using the *cell_explorer* function of *CrystFEL* with the *DIRAX* (Duisenberg, 1992 ▸) or *MOSFLM* (Leslie, 2006 ▸; Powell, 1999 ▸) indexing method. The sample-to-detector distance and indexing parameters were optimized manually so as to improve the width of the unit-cell parameter distributions. The final indexing was performed using the *MOSFLM* method. The optimized sample-to-detector distance was 52.0 ± 0.1 mm, indicating about 0.2% accuracy (Supplementary Fig. S2).

SR diffraction data were collected at 100 K using a MAR Mosaic 225 CCD detector on beamline BL26B2 at SPring-8, Japan. The wavelength used was 1.000 Å and the sample-to-detector distance was set to 200.0 mm. The SR data collected with an oscillation angle of 0.5° were processed and scaled without an intensity cutoff using *HKL*-2000 (Otwinowski & Minor, 1997 ▸). For both SFX and SR, the experimental data including negative intensities were converted to positive-amplitude data based on Bayesian statistics using *CTRUNCATE* (French & Wilson, 1978 ▸) from the *CCP*4 program suite (Winn *et al.*, 2011 ▸). All of the crystal structures were solved and refined using the *PHENIX* program package (Adams *et al.*, 2002 ▸), in which the previously reported SFX structure of thermolysin (PDB entry 4ow3; Hattne *et al.*, 2014 ▸) was used as the search model for molecular replacement. In each cycle of the *PHENIX* refinement except for the last few cycles, the simulated-annealing (torsion-dynamics) protocol was adopted to eliminate model bias. The structure was visualized/revised using *Coot* (Emsley & Cowtan, 2004 ▸). Special care was taken in the water placement, where only a water model with regular electron density not less than 0.5σ in a 2*mF*_o_ − *DF*_c_ map and satisfying the criteria of interatomic interactions (hydrogen bonds with distances not less than 2.2 Å and not greater than 3.4 Å; nonpolar interactions with distances not less than 2.65 Å and not greater than 4.2 Å) was selected by visual inspection in each cycle of the refinement. For the comparison of effective resolutions between data sets, the resolution limit of each data set was adjusted at the last stage of the structure refinement so that the *R*_free_ value for the outmost shell was in the range 20–30%. The statistics from crystallographic analysis are summarized in Table 1 ▸. Structural superposition at corresponding C^α^ atoms was performed using *LSQKAB* (Kabsch, 1976 ▸) in the *CCP*4 suite. The distribution of C^α^ deviations from the superposition analysis was statistically examined by the Mann–Whitney *U*-test (Mann & Whitney, 1947 ▸), which confirmed the correctness of our conclusion from the superposition analysis described in §[Sec sec3]3 (Supplementary Tables S1 and S2). The annealed OMIT maps from *PHENIX* (Adams *et al.*, 2002 ▸) were produced at the same resolution as that used for the refinement of the corresponding structure from all atoms of the final model except for those of ZA; a torsion-dynamics protocol of simulated annealing at temperatures from 2500 to 300 K followed by positional refinement and individual *B*-factor refinement was used.

## 3. Results and discussion   

### 3.1. Quality of the crystal structures   

**Figure 1:**
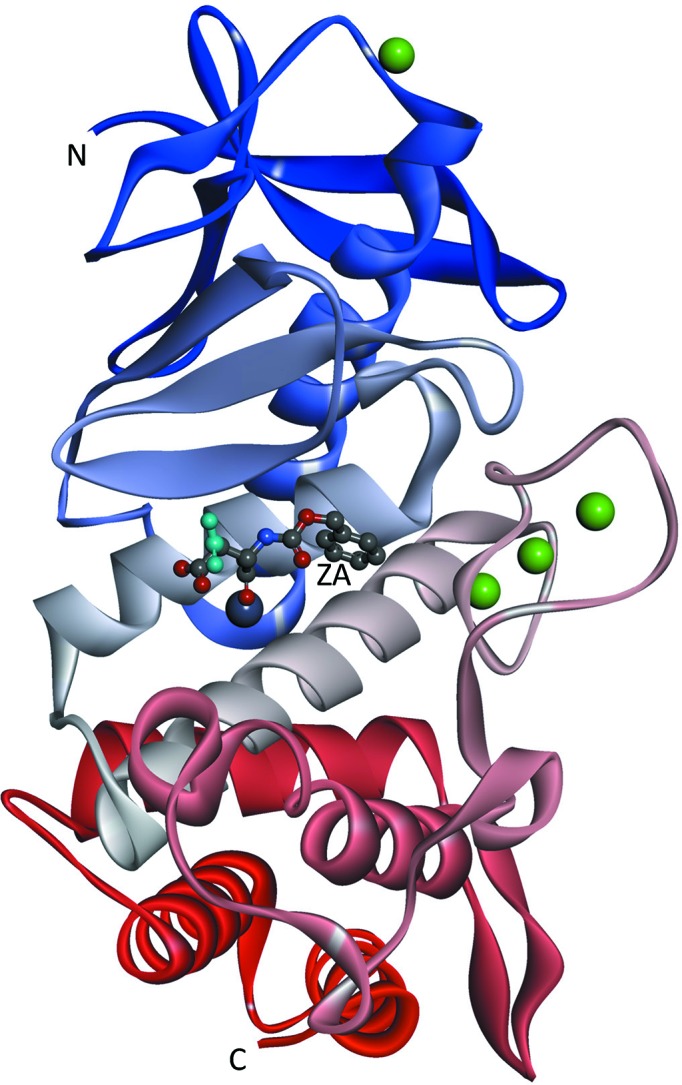
Overall structures of the thermolysin–ligand complex from the liganded oil–SFX form. Thermolysin molecules are shown as a ribbon model coloured from the N-terminus to the C-terminus. Bound zinc and calcium ions are shown as grey and green balls, respectively. The bound ZA molecule is shown as a ball-and-stick model with atom-type colouring, apart from the alternate conformation, which is coloured cyan. This figure was prepared with *Discovery Studio* (Accelrys).

**Figure 2:**
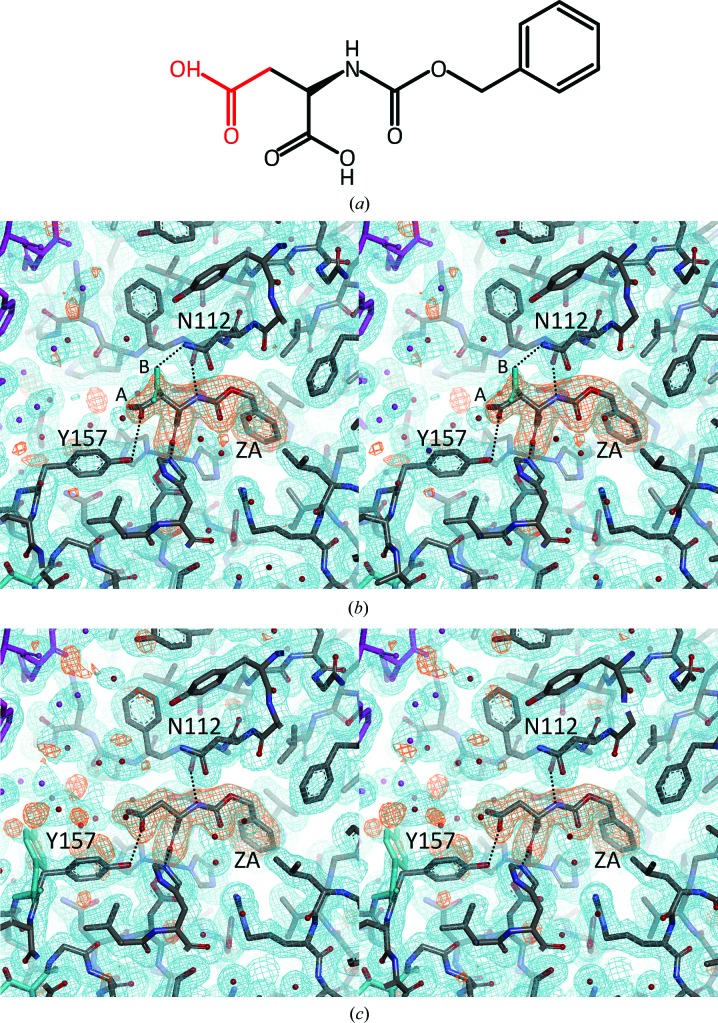
Structural differences in ligand recognition between SFX and SR. (*a*) Chemical structure of the *N*-carbobenzoxy-l-aspartic acid ligand. The carboxymethyl moiety showing alternate conformations is coloured red. (*b*, *c*) Stereo representations of the crystal structure relevant to ligand binding in the liganded oil–SFX form (*b*) and the liganded SR1 form (*c*). Atoms in the asymmetric unit are shown with atom-type colouring, apart from those of the alternate conformation, which are coloured cyan; symmetry-related atoms are coloured magenta. Important residues, the ligand ZA and two alternate conformations of the carboxymethyl moiety of ZA are labelled. Ligand–protein hydrogen bonds are indicated as dotted lines. The annealed OMIT maps for the ligand molecule with Fourier coefficients 2*mF*_o_ − *DF*_c_ (blue; 0.5σ contour level) and *mF*_o_ − *DF*_c_ (orange; 3.σ contour level) are overlaid. (*b*) and (*c*) were prepared with *Discovery Studio* (Accelrys).

Three SFX and two SR structures of thermolysin have been determined at comparable resolutions in the range 1.9–2.3 Å (Table 1 ▸ and Supplementary Fig. S1). The present structures and four previously reported structures (PDB entries 3qgo, 3qh1, 3qh5 and 4ow3; Birrane *et al.*, 2014 ▸; Hattne *et al.*, 2014 ▸) share the same crystal packing; all of the crystals belong to the same space group, *P*6_1_22, with similar unit-cell parameters and contain a thermolysin monomer in the asymmetric unit. The final models of the present thermolysin structures with well defined electron densities contained entire amino-acid residues 1–316, a functional zinc ion at the active site and four structural calcium ions (Fig. 1 ▸). In addition, the liganded SR1 structure contained molecules of polyethylene glycol, which was used as a cryoprotectant. In the liganded forms, all atoms comprising the ligand ZA (Fig. 2 ▸ *a*) were identified in the electron-density map with reasonable *B* values (Table 1 ▸). In the liganded water–SFX form using cellulose as a crystal carrier, one of the four calcium sites had a considerably low occupancy of 0.72, which may be relevant to the calcium-chelating effect of cellulose in the presence of certain carboxylic acids (Rhee & Tanaka, 2000 ▸). The average *B* values calculated from the final models were comparable to the corresponding Wilson *B* values from the diffraction data. Stereochemical analysis in the *PHENIX* program suite (Adams *et al.*, 2002 ▸) revealed no residues in the outlier region of the Ramachandran plot. Probably owing to the limited flexibility of the thermolysin molecule, all of the present structures share essentially the same backbone conformations, with similar patterns of *B*-factor distribution; this is in contrast to previous work on a G-protein-coupled receptor (GPCR) that showed structural differences in certain flexible loops between SFX and SR (Liu *et al.*, 2013 ▸).

### 3.2. Comparison between SFX and SR   

Many experiments have been reported on the thermal contraction of protein crystals at low temperatures using conventional X-ray sources (in-house source and synchrotron radiation; Haas & Rossmann, 1970 ▸; Walter *et al.*, 1982 ▸; Hartmann *et al.*, 1982 ▸; Frauenfelder *et al.*, 1987 ▸; Tilton *et al.*, 1992 ▸; Young *et al.*, 1993 ▸; Keedy *et al.*, 2015 ▸). In agreement with these reports, the unit-cell lengths of the present thermolysin crystals are 0.7–1.6% longer in the SFX structures at 300 K when compared with those in the SR structures at 100 K (Table 1 ▸). This difference is comparable to those in reported experiments at cryogenic (80–100 K) and ambient (298–300 K) temperatures: 1.7–2.4% for myoglobin crystals (Hartmann *et al.*, 1982 ▸), 0.9–2.7% for ribonucrease A crystals (Tilton *et al.*, 1992 ▸), 0.4–2.8% for lysozyme crystals (Young *et al.*, 1993 ▸) and 1.3–1.8% for cyclophilin A crystals (Keedy *et al.*, 2015 ▸). Thus, the unit-cell parameters obtained from our SFX experiments may be correct for those of thermolysin crystals at room temperature. Notably, the unit-cell parameters agree well with each other in the SFX structures. In the SR structures, the difference in the cryoprotection procedure resulted in 0.4–0.7% differences in unit-cell lengths, whereas the difference was within 0.2% in the SFX structures.

Phenomena involving atom displacement such as thermal vibration and alternate conformations can be modulated by the temperature at which the diffraction data were collected. It has been reported that the *B* factors of protein atoms are reduced at low temperatures (Walter *et al.*, 1982 ▸; Hartmann *et al.*, 1982 ▸). In the present structures, the average *B* values are higher for SFX at room temperature, as expected (Table 1 ▸). However, it has been reported that the Monte Carlo integration of still images can produce artificially large *B* factors in SFX (Kroon-Batenburg *et al.*, 2015 ▸). Thus, unfortunately, it is unclear whether or not the larger *B* factors observed in the present SFX structures reflect higher thermal vibration. A restrained conformational fluctuation of protein atoms at low temperatures has also been reported (Keedy *et al.*, 2015 ▸). In the present structures, it is not conclusive whether or not the conformational fluctuation is restrained at low temperature. However, in the SR structures the locations of the alternate conformations that were observed were varied depending on the cryoprotection procedure, whereas they were identical in the SFX structures (Table 1 ▸). When the residues with alternate conformations are compared between the SR and SFX structures, only four of 12 residues were found in common. The SFX structures may more closely represent the physiological mode of the conformational fluctuation at room temperature when compared with the SR structures.

**Figure 3:**
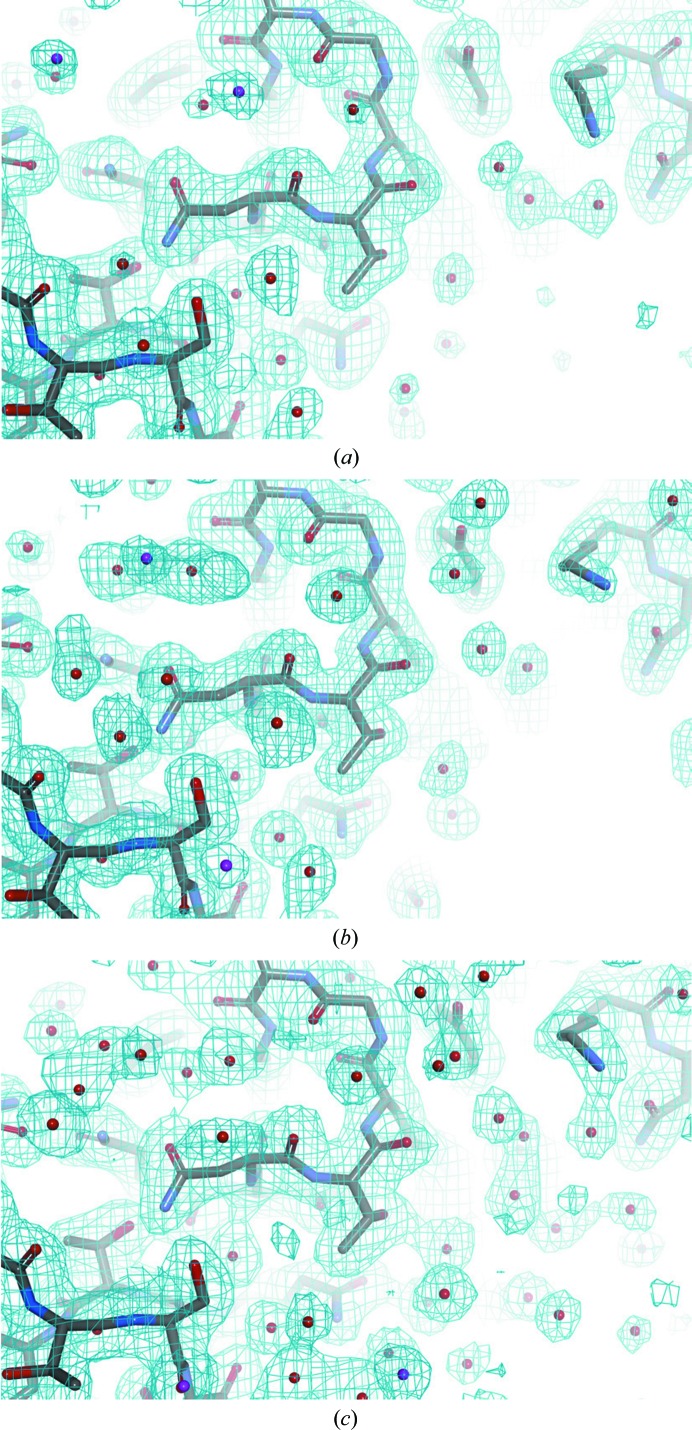
Structural differences in water coordination. Crystal structures delineating part of the water coordination in the liganded oil–SFX form (*a*), in the liganded SR1 form (*b*) and in the liganded SR2 form (*c*) are shown. Water molecules are shown as spheres on the molecular surface of the protein. Atoms in the asymmetric unit are shown with atom-type colouring; symmetry-related atoms are coloured magenta. The final 2*mF*_o_ − *DF*_c_ maps are overlaid with contour levels of 0.5σ for the liganded oil–SFX form, 0.7σ for the liganded SR1 form and 0.6σ for the liganded SR2 form. The figures were prepared with *Discovery Studio* (Accelrys).

Another difference between SFX and SR is in the water structure. An increment in the number of ordered water molecules at low temperatures has been reported in conventional crystal structures (Earnest *et al.*, 1991 ▸; Young *et al.*, 1993 ▸). However, from a statistical analysis of PDB entries, the increase in the number of water molecules at low temperature was not significant owing to a large variation in the ratio between water and protein atoms in the low-temperature structures (Carugo & Bordo, 1999 ▸). In the present work, the water:protein ratio is calculated to be 0.158–0.197 for the SR structures and 0.113–0.119 for the SFX structures (Table 1 ▸). The values for the SFX structures are similar to each other and are close to the statistically predicted value of 0.111 ± 0.004 for a 2.0 Å resolution structure at room temperature (Carugo & Bordo, 1999 ▸), regardless of the type of crystal carrier used. On the other hand, the values for the SR structure are very different from each other depending on the cryoprotection procedure. Furthermore, the values are much higher than the predicted value of 0.114 ± 0.008 for a 2.0 Å resolution structure at low temperature (Carugo & Bordo, 1999 ▸). From visual inspection of the structures, water molecules in the SFX structures are only observed within the first layer of the water coordination shell, in which a water molecule directly interacts with the protein atoms (Fig. 3 ▸ *a*). In particular, in a pair of liganded SFX structures 80–82% of the water molecules can be overlaid at common positions with inter­atomic distances of less than 1.5 Å (Table 2 ▸), indicating high reproducibility of the water structure regardless of the type of crystal carrier used. The percentage of common waters in SFX structures is substantially lower in a pair of unliganded structures at 70–74%, probably reflecting the ligand binding. Of the water molecules in the SFX structures, 67–76% are commonly observed in the SR structures. However, in the SR structures many additional water molecules are observed both in the first and the outer layers of the water coordination shell depending on the cryoprotection procedure (Figs. 3 ▸ *b* and 3 ▸ *c*). The degree of water coordination in the SR structures is highly dependent on the cryoprotection procedure, indicating poor reproducibility of the water structure from SR. In conclusion, SFX may provide a closer representation of the physiological water structure when compared with SR.

### 3.3. Difference in ligand recognition   

From an SFX experiment using thermolysin microcrystals soaked with the small-molecule ligand ZA, a protein–ligand complex structure was successfully obtained (Fig. 1 ▸). Both oil-based and water-based crystal carriers provided identical structures, including the mode of ligand recognition (Fig. 2 ▸ *b* and Supplementary Fig. S1). The enzymatic active site of the thermolysin molecule binds a ZA molecule in place of the substrate. The carboxymethyl moiety of ZA has two alternate conformations: *A* and *B* conformers with occupancies of 0.56 and 0.44, respectively. The *A* and *B* conformers are hydrogen-bonded to the O^η^ atom of Tyr157 and the N^δ2^ atom of Asn112, respectively. On the other hand, an SFX experiment without ligand soaking provided an apoenzyme structure. The apo structure was essentially the same as the reported SFX structure (PDB entry 4ow3; Hattne *et al.*, 2014 ▸), except for subtle conformational differences in the side chains of Asn112, Thr157 and Glu166 with interatomic distances of less than 1 Å between corresponding atoms after C^α^ superposition of two structures (Supplementary Fig. S1). The overall root-mean-square deviation (r.m.s.d.) value from the superposition was 0.203 Å, which was significantly lower than those from the other comparisons of the SFX structures with PDB entry 4ow3 (0.224–0.228 Å), probably reflecting the ligand binding (Table 3 ▸ and Supplementary Table S2). The SR experiment using conventional ligand soaking with ZA also provided protein–ligand complex structures similar to but distinct from the liganded SFX structures (Fig. 2 ▸ *c* and Supplementary Fig. S1). Essentially the same structures were obtained from both of the cryoconditions for the SR experiment, except that two alternate conformations were observed for Tyr157 in the liganded SR1 structure. The ZA molecule had no alternate conformations and was hydrogen-bonded to the O^η^ atom of Tyr157 in the same manner as in the *A* conformer in the liganded SFX structures.

When the present liganded SR structures are compared with the reported cognate structure, PDB entry 3qh1 (Birrane *et al.*, 2014 ▸), the r.m.s.d. values from the C^α^ superposition are 0.135–0.148 Å, which are significantly lower than those from the other comparisons (0.178–0.228 Å), indicating an overall similarity among the liganded SR structures (Table 3 ▸ and Supplementary Table S2). However, at the ligand-binding site of PDB entry 3qh1 the carboxymethyl moiety of ZA is hydrogen-bonded to the N^δ2^ atom of Asn112 in the same manner as in the *B* conformer of our liganded SFX structures, and alternate conformations are observed for Asn112 but not for Tyr157, which is distinct from the present liganded SR structures. Therefore, in terms of the ligand-recognition mode, the three available liganded SR structures differ from each other. In contrast, the same ligand-binding mode was reproducibly observed from the SFX soaking experiments. In fact, the C^α^-superposition analysis could detect the ligand binding in the SFX structures (Table 2 ▸ and Supplementary Table S1); of the r.m.s.d. values from the superposition between the SFX structures, that of 0.057 Å for a pair of liganded structures was significantly lower than those for the other pairs (0.106–0.112 Å). The other comparisons including the SR structures gave much higher r.m.s.d. values in the range 0.158–0.192 Å. Furthermore, the ligand-binding mode observed in the SFX structures was not available from the SR experiments.

The present SFX and SR structures of thermolysin reveal considerable differences in ligand recognition, which is in contrast to the previous work on GPCR, which reported no differences in ligand recognition between SFX and SR structures (Liu *et al.*, 2013 ▸). Differences in the temperature of data collection and in the procedure of cryoprotection are suggested as possible reasons for the structural differences that are observed. In addition, several groups have pointed out structural changes in proteins owing to radiation damage when crystal structures from XFELs and conventional X-ray sources are compared (Hirata *et al.*, 2014 ▸; Suga *et al.*, 2015 ▸; Fukuda *et al.*, 2016 ▸). Thus, certain modifications from radiation damage may be another reason for the present structural differences.

## 4. Conclusions   

In this work, the feasibility of ligand screening in SFX has been examined using thermolysin as a model system. As a result, a ligand-soaking experiment using SFX successfully provided untreated protein–ligand complex structures at room temperature. From a structural comparison between SFX and SR, clear structural differences in the ligand-binding mode were observed. Notably, the SFX structures were highly reproducible regardless of the type of crystal carrier used, whereas the SR structures showed substantial differences depending on the cryoprotection procedure that was used; C^α^ superposition between the liganded SR1 form and the liganded SR2 form provided a distribution with significantly higher values of the C^α^ deviation when compared with any superposition between a pair of SFX structures (Table 2 ▸ and Supplementary Table S1). In conclusion, ligand screening in SFX may be useful for the design of small-molecule ligands for SBDD in the near future, because it provides structural information without factors that may affect the physiological structure of proteins.

## Supplementary Material

PDB reference: thermolysin, SFX, liganded form with oil-based carrier, 5wr2

PDB reference: SFX, liganded form with water-based carrier, 5wr3

PDB reference: SFX, unliganded form with oil-based carrier, 5wr4

PDB reference: SR, liganded form with cryocondition 1, 5wr5

PDB reference: SR, liganded form with cryocondition 2, 5wr6

Supplementary Figures and Tables.. DOI: 10.1107/S2059798317008919/wa5113sup1.pdf

## Figures and Tables

**Table 1:** Statistics from crystallographic analysis Values in parentheses are for the outermost shell.

Sample	Liganded oil–SFX	Liganded water–SFX	Unliganded oil–SFX	Liganded SR1	Liganded SR2
Data collection					
Space group	*P*6_1_22	*P*6_1_22	*P*6_1_22	*P*6_1_22	*P*6_1_22
Unit-cell parameters (Å)	*a* = 93.6, *c* = 131.2	*a* = 93.6, *c* = 131.2	*a* = 93.7, *c* = 131.0	*a* = 92.25, *c* = 129.72	*a* = 92.93, *c* = 129.14
Resolution range (Å)	46.8–2.00 (2.07–2.00)	46.8–2.10 (2.17–2.10)	46.8–2.10 (2.17–2.10)	46.1–1.90 (1.97–1.90)	46.5–2.30 (2.38–2.30)
No. of images: used/collected	17154/37714	17402/41519	4742/14432	148/148	52/52
No. of unique reflections	23565 (2238)	20466 (1874)	20468 (1883)	26366 (2653)	14641 (1435)
Multiplicity	551.8 (83.4)	596.2 (171.2)	137.8 (39.6)	8.6 (6.5)	3.1 (3.1)
Completeness (%)	100.0 (100.0)	100.0 (100.0)	100.0 (100.0)	100.0 (100.0)	96.0 (99.0)
〈*I*/σ(*I*)〉	7.1 (1.5)	9.0 (3.7)	4.8 (2.1)	21.1 (3.8)	10.2 (3.6)
*R*_split_ † (%)	11.0 (64.3)	10.2 (25.8)	20.6 (48.5)	—	—
CC_1/2_ ‡	0.981 (0.538)	0.981 (0.868)	0.923 (0.637)	— (0.833)	— (0.911)
*R*_merge_ § (%)	—	—	—	8.6 (50.3)	11.6 (31.3)
Refinement					
Resolution range (Å)	46.8–2.00 (2.09–2.00)	44.1–2.10 (2.21–2.10)	46.8–2.10 (2.21–2.10)	43.5–1.90 (1.98–1.90)	40.2–2.30 (2.48–2.30)
No. of reflections	23549 (2872)	20453 (2843)	20456 (2865)	26363 (2866)	14639 (2931)
*R*_cryst_/*R*_free_ ¶ (%)	13.2 (25.5)/16.9 (25.8)	12.60 (15.9)/16.0 (20.6)	15.0 (23.1)/18.8 (28.6)	15.4 (20.1)/19.0 (25.7)	15.3 (17.9)/18.2 (24.2)
No. of atoms					
Protein	2432	2432	2432	2432	2432
Ligand	19	19	0	19	19
PEG	0	0	0	26	0
Zinc	1	1	1	1	1
Calcium††	4.00	3.72	4.00	4.00	4.00
Water††	275.56	281.56	290.49	383.65	479.00
Total††	2731.56	2737.28	2727.49	2865.65	2935.00
〈*B*〉 (Å^2^)					
Protein	35.90	28.94	29.32	20.06	19.88
Ligand	37.85	31.70	—	22.00	21.64
PEG	—	—	—	38.36	—
Zinc	31.18	24.80	25.47	15.63	13.68
Calcium	33.69	30.06	27.32	18.73	19.09
Water	52.73	46.73	47.01	39.12	35.32
Total	37.61	30.79	31.20	22.79	22.41
Wilson *B* value (Å^2^)	36.50	31.12	32.20	20.56	20.17
Ratio of water/protein atoms	0.113	0.116	0.119	0.158	0.197
Amino acids in alternate conformations	7	7	7	8	1
Estimated coordinate error‡‡ (Å)	0.15	0.14	0.21	0.17	0.18
R.m.s.d., bond lengths (Å)	0.007	0.007	0.007	0.007	0.012
R.m.s.d., bond angles (°)	1.074	1.032	1.028	1.039	1.254
Ramachandran plot					
Favoured (%)	96.9	96.3	96.6	96.6	96.8
Allowed (%)	3.1	3.7	3.4	3.4	3.2
Outliers (%)	0.0	0.0	0.0	0.0	0.0
PDB code	5wr2	5wr3	5wr4	5wr5	5wr6

† *R*_split_ = 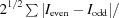 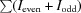, where *I*_even_ and *I*_odd_ represent the intensities of equivalent reflections from even-numbered and odd-numbered images, respectively.

‡ Pearson’s correlation coefficient between averaged intensities of two corresponding observation subsets in which observations of each unique reflection are randomly divided into two half data sets. The programs *CrystFEL* and *HKL*-2000 were used for the SFX data and the SR data, respectively; overall values were not available from *HKL*-2000.

§ *R*_merge_ = 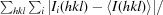 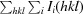, where *I_i_*(*hkl*) is the *i*th observation of reflection *hkl* and 〈*I*(*hkl*)〉 is the weighted average intensity for all observations *i* of reflection *hkl*.

¶ *R*_free_ was calculated using 5% of the reflections that were omitted from refinement.

†† The number of atoms was calculated as the sum of occupancies.

‡‡ The maximum-likelihood-based method in *PHENIX* was used.

**Table 2:** Superposition of the present structures and analysis of common waters A C^α^ superposition was performed between the present structures of thermolysin crystals as shown at the left and top. Amino-acid residues with alternate conformations were excluded from the calculation. The upper value is the r.m.s.d. value of the interatomic distances between corresponding C^α^ atoms after superposition; 304–309 C^α^ atoms were used for the calculation. A statistical examination of the positional differences between the distributions of C^α^ deviations using the Mann–Whitney *U*-test (Mann & Whitney, 1947 ▸) is available in Supplementary Table S1. After the C^α^ superposition, the common water molecules with close interatomic distances of less than 1.5 Å were counted. The number of atoms was calculated as the sum of occupancies. The ratio of the number of common waters to the total number of waters in the structure on the left is shown as the lower value.

	Liganded oil–SFX	Liganded water–SFX	Unliganded oil–SFX	Liganded SR1	Liganded SR2
Liganded oil–SFX		0.057 Å	0.106 Å	0.160 Å	0.182 Å
		81.5%	73.9%	73.9%	74.6%
Liganded water–SFX	—		0.112 Å	0.163 Å	0.182 Å
	79.8%		74.1%	70.2%	75.5%
Unliganded oil–SFX	—	—		0.190 Å	0.192 Å
	70.1%	71.8%		67.0%	71.1%
Liganded SR1	—	—	—		0.158 Å
	53.1%	51.5%	50.7%		67.7%
Liganded SR2	—	—	—	—	
	43.0%	44.5%	43.2%	54.3%	

**Table 3:** Superposition of the present structures with reported structures A C^α^ superposition was performed between the present structures (top) and the reported structures (left). Amino-acid residues with alternate conformations were excluded from the calculation. The r.m.s.d. value of the interatomic distances between corresponding C^α^ atoms after the superposition is shown; 289–313 C^α^ atoms were used for the calculation. A statistical examination (Mann & Whitney, 1947 ▸) of the positional differences between the distributions of C^α^ deviations using the Mann–Whitney *U*-test is available in Supplementary Table S2. R.m.s.d.s are given in Å.

	Liganded oil–SFX	Liganded water–SFX	Unliganded oil–SFX	Liganded SR1	Liganded SR2
4ow3 (unliganded SFX)	0.228	0.224	0.203	0.216	0.210
3qh1 (liganded SR)	0.178	0.182	0.193	0.148	0.135
