# Supplementary material for: Protein–ligand complex structure from serial femtosecond crystallography using soaked thermolysin microcrystals and comparison with structures from synchrotron radiation
Source: Acta Crystallogr D Struct Biol. 2017 Jul 31;73(Pt 8):702–9. doi: 10.1107/S2059798317008919 (PMC5571745; doi:10.1107/S2059798317008919)
Supplement: Supplementary file 1 [file d-73-00702-sup1.pdf]

STRUCTURAL
BIOLOGY

Volume 73 (2017)

Supporting information for article:

Protein-ligand complex structure from serial femtosecond crystallography using soaked thermolysin microcrystals and comparison with structures from synchrotron radiation

Hisashi Naitow, Yoshinori Matsuura, Kensuke Tono, Yasumasa Joti, Takashi Kameshima, Takaki Hatsui, Makina Yabashi, Rie Tanaka, Tomoyuki Tanaka, Michihiro Sugahara, Jun Kobayashi, Eriko Nango, So Iwata and Naoki Kunishima

Supplementary Table S1 Statistical test for the superposition within present structures.

	SFX1–SFX2<br>0.057 Å	SFX1–SFX3<br>0.106 Å	SFX2–SFX3<br>0.112 Å	SFX1–SR1<br>0.160 Å	SFX1–SR2<br>0.182 Å	SFX2–SR1<br>0.163 Å	SFX2–SR2<br>0.182 Å	SFX3–SR1<br>0.190 Å	SFX3–SR2<br>0.192 Å	SR1–SR2<br>0.158 Å
SFX1–SFX2<br>0.057 Å		<0.001	<0.001	<0.001	<0.001	<0.001	<0.001	<0.001	<0.001	<0.001
SFX1–SFX3<br>0.106 Å	<0.001		0.597	<0.001	<0.001	<0.001	<0.001	<0.001	<0.001	<0.001
SFX2–SFX3<br>0.112 Å	<0.001	0.597		<0.001	<0.001	<0.001	<0.001	<0.001	<0.001	<0.001
SFX1–SR1<br>0.160 Å	<0.001	<0.001	<0.001		0.141	0.330	0.061	<0.001	<0.001	0.003
SFX1–SR2<br>0.182 Å	<0.001	<0.001	<0.001	0.141		0.538	0.719	0.002	0.029	<0.001
SFX2–SR1<br>0.163 Å	<0.001	<0.001	<0.001	0.330	0.538		0.308	<0.001	0.003	<0.001
SFX2–SR2<br>0.182 Å	<0.001	<0.001	<0.001	0.061	0.719	0.308		0.006	0.070	<0.001
SFX3–SR1<br>0.190 Å	<0.001	<0.001	<0.001	<0.001	0.002	<0.001	0.006		0.311	<0.001
SFX3–SR2<br>0.192 Å	<0.001	<0.001	<0.001	<0.001	0.029	0.003	0.070	0.311		<0.001
SR1–SR2<br>0.158 Å	<0.001	<0.001	<0.001	0.003	<0.001	<0.001	<0.001	<0.001	<0.001	

The positional differences between the distributions of C^{α} deviations from the superposition analysis within present structures were statistically examined by the Mann-Whitney *U* test (Mann & Whiteney, 1947) using the program *Excel* (Microsoft). The distribution from a superposition between a pair of structures is listed at the top line and the leftmost column with a corresponding r.m.s.d. value as shown in Table 2, using abbreviations of structures as follows: SFX1 for the liganded oil-SFX form, SFX2 for the liganded water-SFX form, SFX3 for the unliganded oil-SFX form, SR1 for the liganded SR1 form, SR2 for the liganded SR2 form. The *U* value from the Mann-Whitney *U* test evaluates an overall positional difference between the two distributions compared. Since each distribution that is assumed to be a parent population has 304–309 data and has similar shape of distribution, *U* values obtained are assumed to be normally distributed. A two-tailed cumulative probability providing the same result or the more extreme results under the null hypothesis of no positional difference between two distributions (*p*-value) was calculated from the corresponding *U* value. The *p*-values obtained are listed in the intersections of the table. For instance, this result confirms following conclusions with a significance level of 0.1%: the C^{α} superposition between the liganded oil-SFX form and the liganded water-SFX form provides a distribution with significantly lower values of C^{α} deviations when compared with any other superposition; a C^{α} superposition between a pair of SFX structures provides a distribution with significantly lower values of C^{α} deviations when compared with any other superposition between any structure and an SR structure; the C^{α} superposition between the liganded SR1 form and the liganded SR2 form provides a distribution with significantly higher values of C^{α} deviations when compared with any superposition between a pair of SFX structures.

Supplementary Table S2 Statistical test for the superposition of present structures with reported structures.

	SFX1–4ow3 0.228 Å	SFX2–4ow3 0.224 Å	SFX3–4ow3 0.203 Å	SR1–4ow3 0.216 Å	SR2–4ow3 0.210 Å	SFX1–3qh1 0.178 Å	SFX2–3qh1 0.182 Å	SFX3–3qh1 0.193 Å	SR1–3qh1 0.148 Å	SR2–3qh1 0.135 Å
SFX1–4ow3 0.228 Å		0.822	0.012	<0.001	0.007	<0.001	<0.001	<0.001	<0.001	<0.001
SFX2–4ow3 0.224 Å	0.822		0.004	<0.001	0.002	<0.001	<0.001	<0.001	<0.001	<0.001
SFX3–4ow3 0.203 Å	0.012	0.004		0.289	0.747	<0.001	0.005	0.054	<0.001	<0.001
SR1–4ow3 0.216 Å	<0.001	<0.001	0.289		0.411	0.039	0.181	0.543	<0.001	<0.001
SR2–4ow3 0.210 Å	0.007	0.002	0.747	0.411		0.002	0.017	0.118	<0.001	<0.001
SFX1–3qh1 0.178 Å	<0.001	<0.001	<0.001	0.039	0.002		0.388	0.109	<0.001	<0.001
SFX2–3qh1 0.182 Å	<0.001	<0.001	0.005	0.181	0.017	0.388		0.455	<0.001	<0.001
SFX3–3qh1 0.193 Å	<0.001	<0.001	0.054	0.543	0.118	0.109	0.455		<0.001	<0.001
SR1–3qh1 0.148 Å	<0.001	<0.001	<0.001	<0.001	<0.001	<0.001	<0.001	<0.001		0.686
SR2–3qh1 0.135 Å	<0.001	<0.001	<0.001	<0.001	<0.001	<0.001	<0.001	<0.001	0.686	

The positional differences between the distributions of C^{α} deviations from the superposition analysis of present structures with reported structures were statistically examined by the Mann-Whitney *U* test (Mann & Whiteney, 1947) using the program *Excel* (Microsoft). The distribution from a superposition between a pair of structures is listed at the top line and the leftmost column with a corresponding r.m.s.d. value as shown in Table 3, using abbreviations of structures as follows: SFX1 for the liganded oil-SFX form, SFX2 for the liganded water-SFX form, SFX3 for the unliganded oil-SFX form, SR1 for the liganded SR1 form, SR2 for the liganded SR2 form. The *U* value from the Mann-Whitney *U* test evaluates an overall positional difference between the two distributions compared. Since each distribution that is assumed to be a parent population has 292–313 data and has similar shape of distribution, *U* values obtained are assumed to be normally distributed. A two-tailed cumulative probability providing the same result or the more extreme results under the null hypothesis of no positional difference between two distributions (*p*-value) was calculated from the corresponding *U* value. The *p*-values obtained are listed in the intersections of the table. For instance, this result confirms following conclusions: the C^{α} superposition between the unliganded oil-SFX form and 4ow3 provides a distribution with significantly lower values of C^{α} deviations when compared with any other superposition between an SFX structure with 4ow3 ($p < 0.05$); a C^{α} superposition between an SR structure with 3qh1 provides a distribution with significantly lower values of C^{α} deviations when compared with any other superposition except for that between another SR structure with 3qh1 ($p < 0.001$).

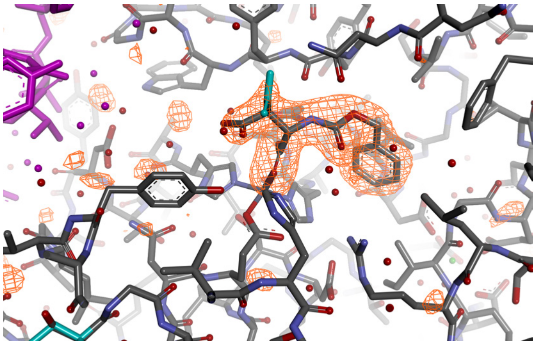

Liganded oil-SFX

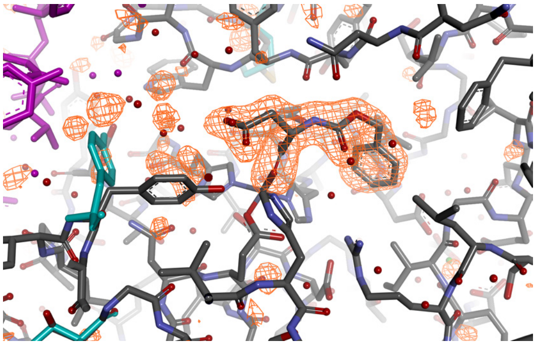

Liganded SR1

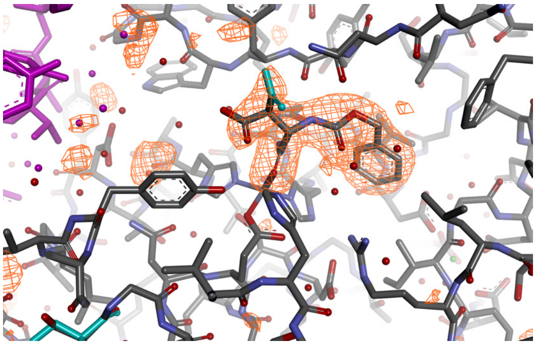

Liganded water-SFX

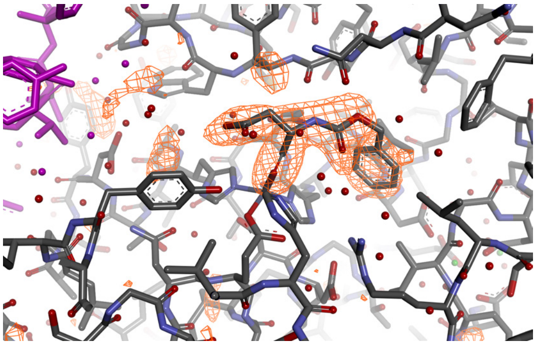

Liganded SR2

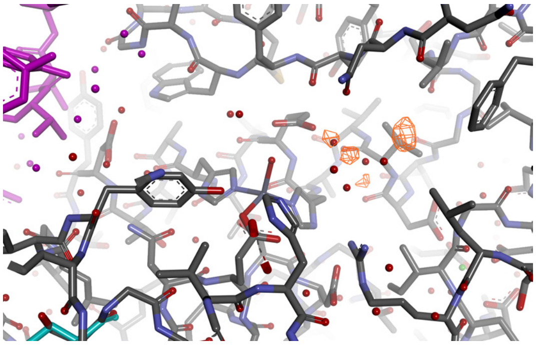

Uniganded oil-SFX

Thermolysin structures in the vicinity of active-site. Atoms in the asymmetric unit are shown with the atom-type coloring except that those of the alternate conformation are colored cyan; the symmetry-related atoms are colored magenta. For the liganded forms, mF_o-DF_c annealed omit maps for the ligand molecule are overlaid with a contour level of 3.0 σ. For the uliganded oil-SFX form, a final mF_o-DF_c map is overlaid with a contour level of 5.0 σ. This figure was prepared with *Discovery Studio* (Accelrys Inc.).

Supplementary Figure S2 Naitow *et al.*

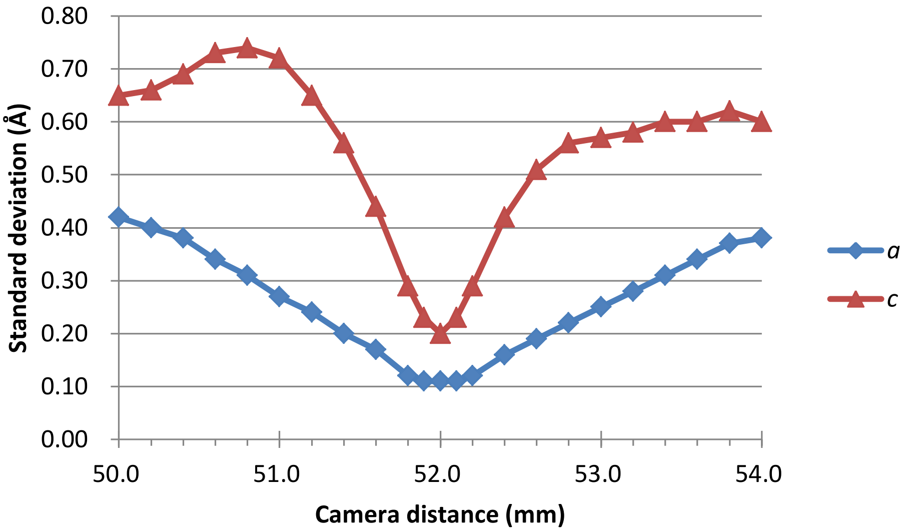

Optimization of sample-detector distance. The sample-detector distance was optimized manually so as to improve the width of cell-parameter distributions. The SFX data were processed using the program *CrystFEL* ver.0.6.0 (White *et al.*, 2012) with the index method of *MOSFLM*. On the liganded oil-SFX form, the standard deviation of a cell-parameter distribution from the cell_explorer function of *CrystFEL* was plotted versus the camera distance. The data for the *a* axis and for the *c* axis are shown as blue diamonds and red triangles, respectively. The sample-detector distance optimized was 52.0±0.1 mm, indicating about 0.2% of accuracy.
